# New R-Based Methodology to Optimize the Identification of Root Endophytes against *Heterobasidion parviporum*

**DOI:** 10.3390/microorganisms7040102

**Published:** 2019-04-06

**Authors:** Linda Rigerte, Kathrin Blumenstein, Eeva Terhonen

**Affiliations:** Forest Pathology Research Group, Büsgen-Institute, Department of Forest Botany and Tree Physiology, Faculty of Forest Sciences and Forest Ecology, University of Göttingen, Büsgenweg 2, 37077 Göttingen, Germany; lindarigerte@gmail.com (L.R.); kathrin.blumenstein@uni-goettingen.de (K.B.)

**Keywords:** fungal endophytes, inhibitory activity, biocontrol, forest pathogens, conifers

## Abstract

Many root fungal endophytes inhabiting forest trees have potential impact on the health and disease progression of certain tree species. Hence, the screening of root endophytes for their biocontrol abilities is relevant for their potential to protect their hosts against invaders. The aim of this research is to screen for the potential inhibitory effects of selected conifer root endophytes during interaction, in vitro, with the root rot pathogen, *Heterobasidion parviporum*. Here, we introduce a guideline that facilitates the use of root fungal endophytes as biocontrol agents. We isolated fungal root endophytes from eight different conifers. These root fungal endophytes were evaluated for their antagonism against the root rot pathogen, *H*. *parviporum*, by means of paired-culture antagonism assays. We determined the antagonism of the isolated root fungal endophytes to elucidate potential biocontrol applications. For the analysis, a software package in R was developed. Endophyte candidates with antagonistic potential were identified.

## 1. Introduction

Endophytes are organisms that, at some stage in their lifecycle, colonize a portion of a plant’s tissue internally without causing any visible disease symptoms [1,2,3,4]. The increasing interest in plant endophyte communities is derived from their apparent potential to positively influence stress tolerance in trees, further providing new sources to be exploited in tree health protection [5]. To understand the function of root endophytes in forest trees, inoculation studies on host trees have been performed. The observed effects vary from beneficial interactions [6,7,8,9,10], to neutral [11], and sometimes even to pathogenic [8,12]. The nature of root endophyte–host interactions seems to depend on the strain of endophyte and the experimental environment [12,13,14,15]. In the context of forest pathology, the most beneficial aspect of endophytes is the production of unique secondary metabolites, which may limit pathogen growth [6,7,8,16,17]. Tellenbach and Sieber [6] isolated compounds from *Phialocephala europaea*, of which, sclerin and sclerotinin A were demonstrated to significantly reduce the growth of *Phytophtora citricola sensu lato*. Similarly, Terhonen et al. [8] found that metabolites extracted from liquid cultures of root endophytes, *Phialocephala sphareoides* and *Cryptosporiopsis* sp., inhibit the growth of several plant pathogens (*Heterobasidion parviporum*, *Phytophtora pini*, and *Botrytis cinerea*). Fungal endophytes can also decrease the infections of pathogens in their host roots, which can be observed in a study conducted by Tellenbach and Sieber [6], who showed that some strains of *Phialocephala subalbina* could reduce disease severity caused by the two oomycete root rot pathogens, *Elongisporangium undulatum* and *Phytophtora plurivora*, in Norway spruce seedlings. Terhonen et al. [8] demonstrated that the root endophyte, *P*. *sphareoides*, was able to prevent the infections of Norway spruce seedling roots by the pathogen, *H*. *parviporum*, in vitro. Indeed, fungal endophytes might play an important role in ecosystem dynamics, affecting not just the fitness of the specific host plant, but also the overall structure and well-being of tree communities [5]. This is supported by the fact that entire assemblages of endophytes have adapted to specialized habitats along with their hosts; for example, aquatic fungi that inhabit submerged plant roots [18].

The dominating group of root endophytes in tree roots are the ‘dark septate endophytes’ (DSEs) [15,19,20,21,22,23,24,25,26,27]. DSEs feature a very characteristic darkly pigmented (“melanized”) hyphal growth inside roots [9,16]. The *Phialocephala fortinii* s.l.–*Acephala applanata* species complex (PAC) is the dominant group of DSEs and they are among the best-characterized DSEs [15,25,28,29,30,31,32]. Members of the PAC species complex are cryptic and they are very widespread and abundant in roots of conifers and ericaceous plants [15,19,20,23,24,25,27]. Generally, DSEs do not seem to have host specificity; however, there are some species that seem to prefer certain host taxa and some of the PAC species preferentially occur on the Pinaceae family [15,19,20,21,24,33,34].

*Heterobasidion annosum sensu lato* (s.l.) is the main cause of the root rot of conifers in Europe [35,36,37,38]. The *H*. *annosum* s.l. complex in Europe consists of three native species: *H*. *annosum sensu stricto*, *H*. *parviporum*, and *H*. *abietinum* [35,39,40]. Of these, *H*. *parviporum* is the major pathogen for the economically important conifer, Norway spruce (*Picea abies*). *H*. *parviporum* infects the tree through deposition of airborne basidiospores on freshly cut stumps, where infection can easily initiate [41,42,43]. As the infection progresses, wood material is rapidly colonized by *H. parviporum*. After the infection is established, *Heterobasidion* species can remain viable in these stumps for decades [44], resulting in an inoculum source for new tree generations [43,44,45] by spreading through root contacts to neighbouring trees [45,46]. In addition, as *H. parviporum* can remain undetected in the host tree for many years, the risk of *H. parviporum* infecting neighbouring healthy trees via root contacts increases [43,44,45,46]. As such, in search of control measures against *H. parviporum*, one interesting direction to pursue is the study of root endophytes. The possible inhibitory effect of root endophytes on root pathogens, such as *H*. *parviporum*, can be harnessed for potential biocontrol applications. These kinds of fungi could be used in infected sites to protect the roots of newly planted seedlings. Terhonen et al. [19] demonstrated that 17% of root endophytes isolated from a spruce-dominated site are capable of inhibiting the growth of *H*. *parviporum*. Particularly, the root endophyte, *P*. *sphareoides*, not only inhibits the necrotrophic *H*. *parviporum*, but also promotes host growth and health [8]. However, the ecological and potential functional role of these abundant endophytic fungi in the host-endophyte interaction remains largely unexplored.

In this study, the root fungal endophytes of eight conifer tree species from the Forest Botanical Garden of the University of Göttingen (Germany) were isolated and tested for their antagonistic reaction against *H*. *parviporum*. The identities and phylogeny of the isolated root endophytes were established on the basis of their internal transcribed spacer (ITS) region sequences. The antagonism of the endophytes to *H*. *parviporum* was investigated by dual culture assays on agar media. This work aims to determine the antagonism of the isolated root endophytes and elucidate potential biocontrol applications within the contextual constraints described in the sections above. It also aims to evaluate the potential inhibitory effect of a subset of the isolated endophytes on the pathogen, *H*. *parviporum*. To analyse this, a software package in R was developed.

## 2. Materials and Methods

Fine root samples were collected in early January 2018 from mature conifer trees with no visible disease symptoms in the Forest Botanical Garden, Göttingen, Germany (51°33′26.55″ N/9°57′40.72″ E). A simple random sampling pattern was applied for tree selection to minimize sampling bias. Fine roots from conifer hosts exhibiting no visible signs of mycorrhizae were collected, placed in Falcon tubes, and stored at −20 °C. Eight different conifer species were sampled. Overall, only one tree was sampled each for *Picea pungens*, *Picea sitzenchis*, and *Pinus peuce*, two trees each for *Picea omorika*, *Picea glauca*, and *Pinus jeffreyi*, and three trees each for *P. abies* and *Pinus sylvestris* (Table 1).

The collected fine roots were processed in 24 h. First, they were washed under running tap water to remove soil particles and cut into small pieces. Root segments were disinfected in 99% ethanol (C_2_H_5_OH) for 1 min, 2% sodium hypochlorite (NaOCl) for 3 min, followed by 70% ethanol for 1 min; the roots were then rinsed in autoclaved ultrapure water H_2_O for 3 to 5 times. The surface sterilized roots were placed on 1.5% malt agar plates (MEA) and Modified Melin–Norkrans medium (MMN) plates [47]. The MMN plates were composed as follows: 0.066 g CaCl_2_X2H_2_O (calcium chloride dihydrate), 0.025 g NaCl (sodium chloride), 0.150 g MgSO_4_X7H_2_O (magnesium sulfate heptahydrate), 0.500 g NH_4_(H_2_PO_4_) (ammonium dihydrogen phosphate), 0.500 g KH_2_PO_4_ (potassium dihydrogen phosphate), 0.5 mL FeCl_3_X6H_2_O (iron(III) chloride hexahydrate), 0.200 g C_6_H_12_O_6_ (glucose), 15.000 g agar, and 0.5 mL thiamine-HCl dissolved in 1000 mL distilled water (pH of 5.5–5.8). Altogether, three short root tip segments from each sample tube were placed in MEA and MMN plates. The plates were incubated in darkness at 19 °C with 75% relative humidity. The incubation period necessary for the growth of various root endophytes differed between plates, ranging from 14 days to one month. When the growth of a root endophyte was observed, a small piece of the hyphae was transferred as a pure culture. Sixty-five (65) root endophytes were isolated, and grouped together based on their morphological features to 19 different groups; 1 to 3 members of each group were then chosen for molecular identification (28 samples).

DNA of fungal endophytes was extracted from 150 mg of the homogenized mycelium sample using the “innuPREP Plant DNA Kit” (Analytik Jena AG, Jena, Germany), according to the manufacturer’s instructions. Taq DNA polymerase (Qiagen, Hilden, Germany) was used for PCR amplification of ITS regions with the primer pair, ITS1-F and ITS4 [48]. Briefly, the PCR protocol was as follows: 1X CoralLoad PCR Buffer, 200 µM dNTP, 0.5 µM primer 1, 0.5 µM primer 2, 100 ng template DNA, and 0.2 U/µL DNA polymerase; the reaction was adjusted to 25 µL with autoclaved MQ H2O. Thermocycler parameters were as follows: Initial denaturation at 95 °C for 3 min, followed by 15 cycles of: denaturation at 95 °C for 30 s, annealing at 55 °C for 1 min, extension at 72 °C for 1 min; 15 cycles of: denaturation at 95 °C for 30 s, annealing at 63 °C for 1 min, extension at 72 °C for 1 min; final extension at 72 °C for 10 min and held at 4 °C indefinitely. The quality of the obtained PCR products was checked on 1% agarose gel (with GelRed nucleic acid stain) under UV illumination. Sequencing was performed with ITS4 primer at Microsynth SEQLAB (Göttingen, Germany). For 24 samples, the full coverage of the ITS1-5.8S-ITS2 region was achieved.

The ITS1 and ITS2 regions of all sequenced samples were extracted using the ITSx program [49]. New sequences consisting of ITS1 to ITS2 regions were constructed—concatenation was performed using the sequence concatenation tool available in MEGA X [50]. Sequence identity was established through a search for homology using the National Center for Biotechnology Information’s (NCBI) Basic Local Search Alignment Tool (BLAST) [51]. The top three matches for each sequence were selected based on query coverage (QC) and identity (Mi%) with regional proximity acting as a tiebreaker in case of conflicts. Of these, only the match that had the best coverage and identity was selected as the homolog establishing positive identification of the sample. The homologous sequences were downloaded as an unaligned FASTA file. The concatenated ITS1 to ITS2 regions and their BLAST matches were aligned using MAFFT [52]; alignment parameters were determined automatically by the program (--auto command). The final dataset consisted of 41 nucleotide sequences (24 samples, 16 references, and 1 outgroup sequence) with 635 positions (inclusive of gaps). The Kimura 2-parameter model [53] with gamma distributed rates (5 rate categories; G = 1.915) and a proportion of invariant sites (*I* = 0.032) was selected to model the evolution of the sequences on the basis of its lowest negative log likelihood score (−lnL = −4987.465) in comparison to the evaluated fits for all other models available in MEGA X (evaluated using MEGA X’s internal model selection tool). The initial tree(s) for the ML phylogeny was/were obtained automatically by applying Neighbor-Joining (NJ) and BioNJ algorithms to a matrix of pairwise distances estimated using the maximum composite likelihood (MCL) approach, and then selecting the topology/topologies with superior log likelihood value(s). Molecular phylogeny was established by means of maximum likelihood (ML) analysis with 1000 bootstrap replicates in MEGA X [50,54]. Final adjustments to the generated ML tree were performed in TreeGraph2 [55] and Microsoft PowerPoint. The ITS1 sequences used in BLAST searches were deposited to GenBank with the following accession numbers, MK589294-MK589317.

**Figure 1:**
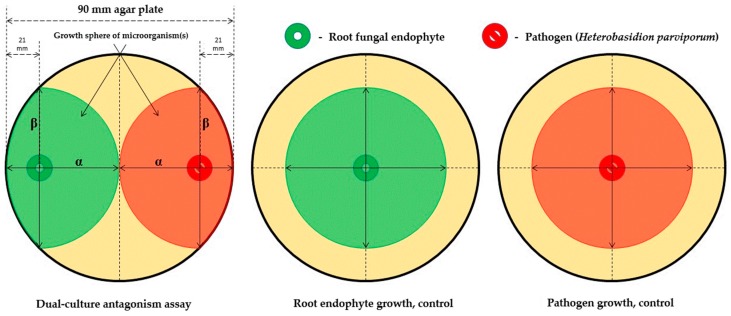
Schematic overview of the setup of the antagonism assay. The dual-culture antagonism assay tests the inhibitory effect of the root fungal endophyte on the pathogen, *Heterobasidion parviporum*; the inhibitory effect is reflected in spherical index (α/β) of the respective organisms. Solitary cultures of the respective root endophyte and the pathogen were also plated—and observed—as controls in this experiment.

The antagonism of the isolated root endophyte against the pathogen, *H. parviporum*, was investigated by means of a paired-growth assay. The ability of a root endophyte to antagonize the pathogen was determined based on the inhibition level over a given period of time. This was achieved by assessing and measuring the concurrent growth of both the endophyte and the pathogen simultaneously on a shared 1.5% MEA nutrient media surface (Figure 1).

**Figure 2:**
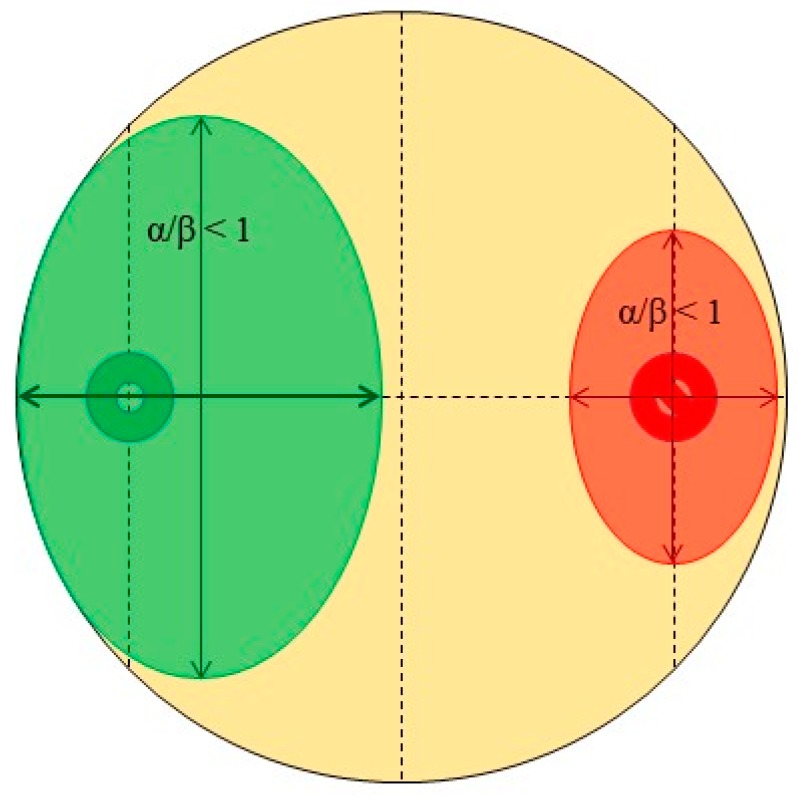
Example schematic antagonism assay plate where both the endophyte antagonist (green) as well as the pathogen (red) have a spherical index < 1.

The antagonistic tests were conducted in the form of dual-culture assays pairing a root fungal endophyte with the pathogen (Figure 1). The root endophyte and pathogen were placed colinear on the surface of malt agar plates (pH = 6) within a distance of 2.1 cm from the perimeter of the petri dish. Such pairings were generated in triplicates for statistical rigor. For each pair of endophyte and pathogen, a control plate containing a single solitary endophyte (spotted identically on the plate as to the paired setup) was also prepared as the endophyte growth control; each endophyte received three controls. Control plates to monitor the pathogen’s growth were also prepared in an identical manner (12 replicates in total). All these plates were then incubated under the same conditions to stimulate the growth of the resident microorganisms (darkness, humidity 75%, 19 °C). Growth measurements were taken 3, 7, and 10 days after inoculation. Measurements were performed for both the root endophytes as well as the pathogen in the antagonism test plate (Figure 2). All isolated endophytes participated in the antagonism assay(s). The measured parameters were the major (α) and minor (β) axes of progression along the colinear axis joining the original spotting locations of the endophyte and/or pathogen. In theory, α measures the progression of the antagonists towards one another (and conversely indicates a measure of the antagonistic effect) while β measures the orthogonal progression (i.e., to α) of the antagonists over the available surface area (an indirect measure of competitive spread). The ratio, α/β, is the spherical index, which is considered a measure of the antagonism exhibited by the endophyte and pathogen against one another [56,57]. The reasoning is that an organism growing in isolation on a circular surface would grow uninhibited radially outwards from the site of inoculation—this corresponds to a spherical index of 1 (since α = β). Therefore, should an inhibitory source be present, radial progression in that particular direction would be negated or minimized, and the spherical index would no longer equal 1 (since α ≠ β). In this regard, a spherical index > 1 (for the endophyte) indicates that the endophyte is inhibiting the pathogen, and an index < 1 indicates it has minimal or no inhibitory effect. Conversely, a spherical index < 1 for the pathogen indicates that it is being (successfully) antagonized by the endophyte, while a spherical index > 1 indicates the endophyte has no effect on the pathogen’s growth or existence. This data included observations under the aforementioned variables for unique endophyte samples (*n* = 65) at every point of measurement (3, 7, and 10 days, respectively) for a total of 195 measurements. This resulted in a large raw dataset to be screened for the identification of successful fungal antagonists to *H. parviporum*. Thus, a filtering process was necessary, and data filtering pipeline was implemented in R to mitigate this challenge; the filtering logic as well as the pipeline are briefly described below.

The explanation provided earlier in this section concerning the spherical index of the root endophyte or the pathogen alone, as a determinant of the fungus’ antagonistic effectiveness against the pathogen, is a naïve interpretation of this number. The spherical index is a ratio resulting from measurements specific to the organism only (and not its surroundings or context), which cannot provide reliable information on the effects of biotic or abiotic factors upon the test organism (nor vice versa) even with replication and statistical testing. For example, consider the case presented in Figure 2: If successful antagonism were indicated only by the spherical index of the endophyte, then this would be an unsuccessful case; conversely, if it were indicated by the spherical index of the pathogen only, then this would be considered a successful case. In truth, it is neither, as neither the antagonist nor the pathogen have grown considerably towards one another. The problem lies in the fact that even under the best-optimized control conditions, biological organisms do not grow evenly nor predictably. In this context, the only way to distinguish a case of an actual, successful antagonism from a spurious one is by considering the spherical ratios of both the antagonist endophyte as well as the pathogen. It can be stated that true antagonism in the experimental conditions described in this section only occurs when the spherical indices indicate that the growth of the endophyte antagonist along the colinear axis (joining the locations of the inoculates of the antagonist and the pathogen) is greater than its growth orthogonal to the colinear axis and also the growth of the pathogen along the colinear axis is less than its growth orthogonal to this axis.

**Figure 3:**
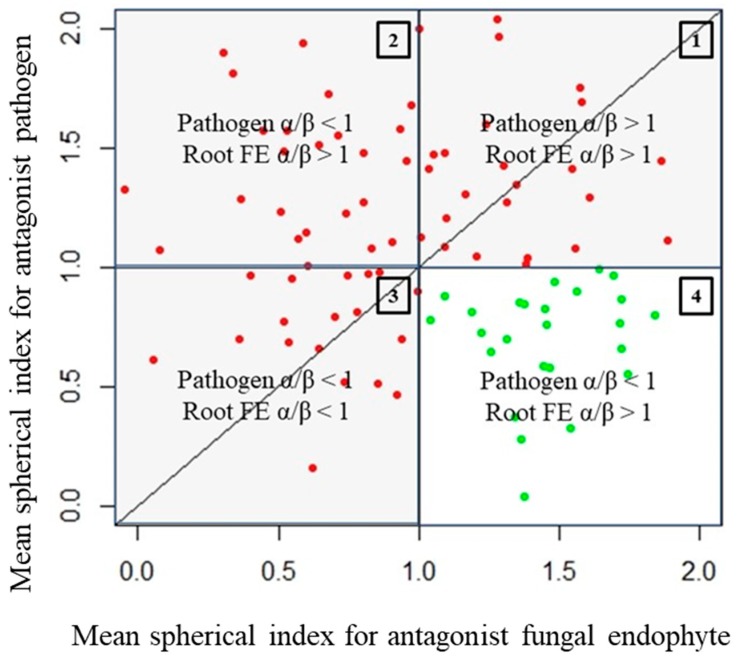
A graph of the mean spherical indices of a set of root fungal endophyte (FE) antagonists plotted against their single test pathogen. This is a simulated dataset generated from a random distribution (*n* = 100, mean = 1, standard deviation = 0.5) that mimics empirical data observed in this experiment. In the graph above, the data points coloured green are non-spurious instances of successful antagonistic interaction while the red data points indicate spurious ones.

This is illustrated in Figure 3 using simulated data (mimicking actual observations). Each data point presented in Figure 3 corresponds to a spherical index pair (X-axis—spherical index of root endophyte, Y-axis—spherical index of pathogen). In Figure 3, the logic described in the paragraphs above corresponds to the unshaded quadrant occupied by the green coloured data points in Figure 3 (pathogen, α/β < 1 and endophyte, α/β > 1; quadrant 4). The data points of every other quadrant can be considered spurious in the context of them being indicative of antagonism from the endophyte to the pathogen.

The reasoning is as follows:

1. Quadrant described by pathogen, α/β > 1, and endophyte, α/β > 1 (quadrant 1): There is clearly no antagonism occurring in these samples as the growth of neither organism along the colinear axis appears to be suppressed or prohibited.
2. Quadrant described by pathogen, α/β > 1, and endophyte, α/β < 1 (quadrant 2): The root endophytes described by their datapoints in this quadrant likely have no effect on the growth of the pathogen (or may conversely even be getting suppressed by the pathogen themselves) as its progression along the colinear axis is higher in comparison to its orthogonal spread and also higher in comparison to the progression of the antagonist along the colinear axis itself.
3. Quadrant described by pathogen, α/β < 1, and endophyte, α/β < 1 (quadrant 3): There is likely no interaction at all between the root endophytes and the pathogen in the cases described by this quadrant as both spherical indices are less than 1; this likely indicates purely natural growth with no antagonistic effects taking place.

Thus, for analysis of this dataset, a short filtering pipeline was constructed in R to select only those samples that met the above-mentioned criteria for consideration as instances of true antagonism (pathogen α/β < 1 and endophyte α/β > 1). The code for the pipeline as well as the statistics are presented in the appendix (Supplementary Files 1 and 2). The means and standard deviations of the pathogen spherical indices and the endophyte spherical indices (‘PRabMean’, ‘PRabSD’, ‘FRabMean’, and ‘FRabSD’ of the 3 replicates each) were first calculated and fed into this pipeline to filter out observations that did not match the selection criteria (pathogen α/β < 1 and endophyte α/β > 1). The original raw dataset was then subdivided into subsets consisting of raw observations (i.e., individual replicate-level observations) from day 3, day 7, and day 10, respectively (Supplementary File 2), consisting of only the selected observations, which were then used for comparative statistics. Comparative statistics involved the 12 pathogen control replicates and the triplicate measurements of the pathogen’s spherical index from the antagonism assays—since the data had already been filtered for spurious measurements, this comparison would be strongly indicative of the presence of actual antagonist endophytes in the testing regime. Comparisons were achieved by means of an unpaired t-test accounting for inequality in the variances of the sample populations (as sample sizes were unequal). The confidence interval for the test statistic was set to 95%. The means of the spherical indices of different groupings of the test data were then tested against the controls. The groupings were: All root endophytes common to a specific tree host, and only the triplicates of individual endophytes.

The null hypothesis was formulated as follows: The difference between the means of the spherical indices of pathogens under antagonism (S1) and the means of the spherical indices of pathogen controls (S2) is zero (i.e., S1–S2 = 0). The alternative hypothesis was formulated as follows: The difference between the means of the spherical indices of pathogens under antagonism (S1) and the means of the spherical indices of pathogen controls (S2) is less than zero (i.e., S1–S2 < 0). The rationale for assuming this alternative hypothesis was that the spherical index of the pathogen under antagonism would be less than one (<1) while the controls, having grown in the absence of any biotic/abiotic pressure(s), should have a spherical index ≈1. Thus, if the effect (i.e., antagonism from that specific endophyte) is real, then the means would also record the same behaviour, and the subtraction of the means of controls from the pathogen replicates involved in the test would then be a negative number (i.e., less than 0). The results of the statistical analysis were then evaluated in the context of the other findings obtained in this study.

## 3. Results

### 3.1. Identification and Diversity of Root Endophytes

A total of 65-root endophytes were obtained from eight coniferous hosts and grouped to 19 different groups based on their morphological differences (Table 2 and Table 3). Only *P. pungens* did not yield any isolates.

**Figure 4:**
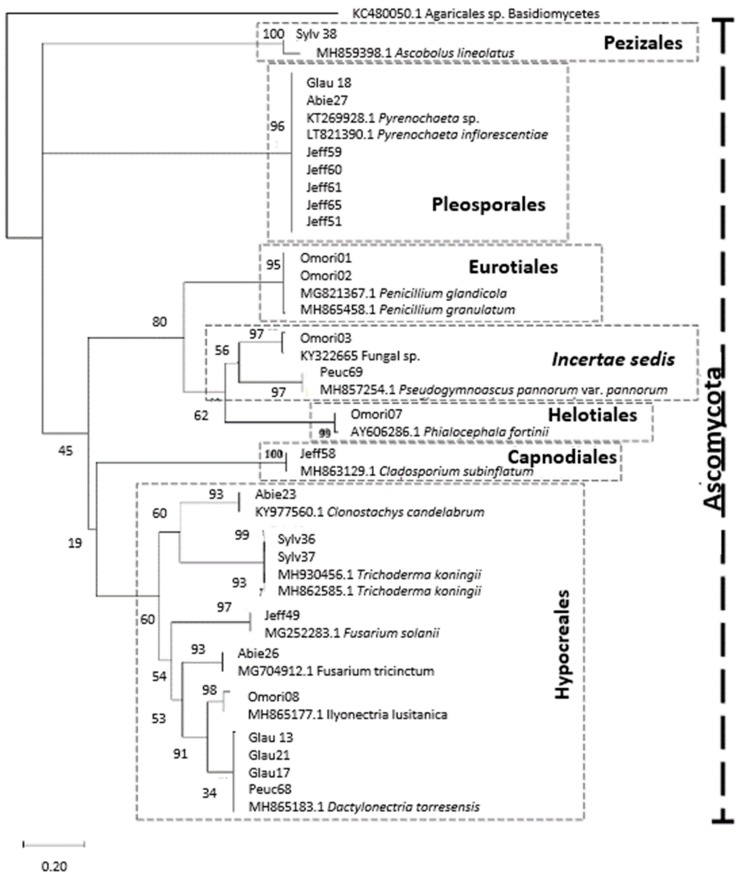
Molecular phylogenetic analysis by maximum likelihood (ML) based on concatenated ITS sequences. The percentage of replicate trees in which the associated taxa clustered together in the bootstrap test are shown next to the branches. Also indicated are the orders to which the samples belong to (dashed rectangles w/descriptions), the phylum (dashed vertical bar), and the outgroup taxon (the Basidiomycete KC480050.1 Agaricales sp. isolated from *P. abies* by Terhonen et al. [19].

The Helotiales sample (Omori07) was identified to be a member of the *Phialocephala fortinii* s.l.—*Acephala applanata* species complex (PAC). The sample, Peuc69, was positively identified as a *Pseudogymnoascus* sp. (100% query coverage and 100% concatenated ITS sequence similarity), but the precise placement of this taxon within the class, Ascomycetes, remains unresolved (Incertae sedis). Although the sample, Sylv38, only had relatively low query coverage and sequence similarity (99% and 97%, respectively, Table 3), potentially being identified as Ascobolus sp., the ML tree firmly established it as belonging to the Pezizales clade (Figure 4). The placements of the root endophytes from the various host trees within the phylogenetic space appears to be freely intermixed, with no readily apparent preferential patterns visible.

The phylogeny was well-resolved up to the species level (Figure 4), with the nodes identifying species relatives featuring strong bootstrap support. Resolution was poorer at the higher taxonomic levels, as the node support was weaker, but yielded 13 distinct clades in total (i.e., lower bootstrap values, Figure 4). Root fungal endophytes belonged to the phylum, Ascomycota, and to the classes, Ascomycetes, Dothideomycetes, Eurotiomycetes, Leotiomycetes, Pezizomycetes, and Sordariomycetes (Table 2). A majority of the identified taxa fell under the orders, Hypocreales and Pleosporales (Table 2, Figure 4). Two taxa were identified as belonging to Eurotiales. One taxa in the orders, Capnodiales, Helotiales, and Pezizales, was also identified.

### 3.2. Antagonisms Assay

The antagonism assay identified 26 of the 65 root endophytes as being statistically successful antagonists against the pathogen, *H. parviporum* (Table 4). None of the assayed samples displayed statistically significant antagonism on day 3. Half the successful samples (13/26) displayed statistically significant antagonism on day 7, and 19/26 samples displayed statistically significant antagonism on day 10. Only six samples showed statistically significant antagonism on both days 7 and 10. These samples were *Dactylonectria* sp. (Glauc15), *Pyrenochaeta* sp. (Glauc18 and Sitch48), Fungal sp. (Omori03), Fusarium sp. (Jeff50), and unknown (Sitch46). *Pinus jeffreyi* was the host with the largest number of successful antagonist root endophytes (9 samples), followed by *Pinus sitchensis* (6 samples), *Picea omori* (5 samples), *Picea glauca* (4 samples), and *Picea abies* (2 samples) (Table 4).

**Figure 5:**
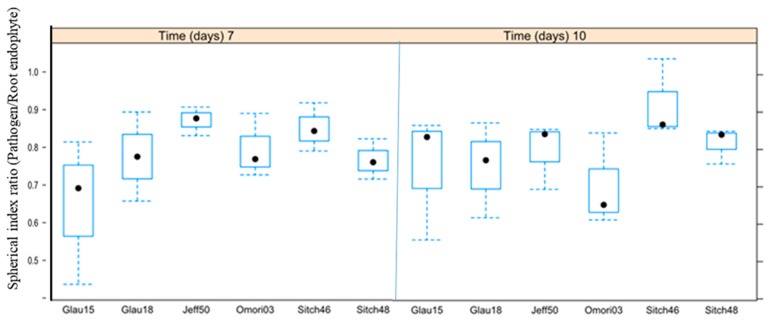
Ratios of the mean spherical indices of the pathogen over its antagonistic partner from day 7 and day 10 measurements of the antagonism assay visualized side-by-side in box-and-whisker plots (solid black circles—medians). Samples shown here are only those which displayed statistically significant antagonism on both days of measurement.

Assuming that smaller values of α/β (i.e., α/β → 0) for the pathogen indicate more successful root endophyte antagonists (Figure 3 and Figure 5), the endophyte, *Cladosporium* sp. (Jeff56), from the host, *Pinus jeffreyi*, is the most successful antagonist in the sample set 7 days after the start of the experiment (pathogen spherical index 0.74 ± 0.13, endophyte spherical index 1.04 ± 0.03). Similarly, all averages of the spherical indices of all antagonists’ root endophytes with statistically significant effects against the pathogen, *H*. *parviporum*, were identified with the R-script (Supplementary Files 1 and 2). To study the temporal stability of the antagonism of samples that yielded statistically significant results on both days 7 and 10, the ratios of their mean spherical indices (i.e., MEAN (pathogen spherical index)/MEAN (endophyte spherical index) for every sample) from both time points of measurement were plotted and visualized (Figure 5). 

## 4. Discussion

Fungal root endophytes could not be isolated from *P*. *pungens*. It is unlikely that this conifer species hosts no endophytes. Hence, it is plausible to suggest the presence of uncultivable root endophytes in this particular species, by which the methodology used in this study is unable to recover them [58,59]. This experimental constraint is consolidated by the fact that no relevant information on the abundances and diversity of this conifer’s root fungal endophyte community is currently available in the literature. The overall community composition observed in this study greatly differs from other studies of conifer roots [19,20,24,60,61]. This study encountered only a single PAC species (from *P*. *omorika*, sample Omori07). This contrasts with previous studies that assume that those DSEs—especially members of the PAC—are predominant and abundant among coniferous hosts [15,19,20,21,24,31,33,34,62]. This result is of special relevance since no PAC species were recovered from well-studied hosts, such as *P*. *abies* and *P*. *sylvestris*. Taking into account that the samples in this study were collected from a non-forest environment, a botanical garden, one explanation could be that the environment influences the composition of endophytic assemblage [63,64]. However, the methodological approach used for the assessment of endophytes’ antagonism against *H*. *parviporum* is methodologically robust. This enables the identification of endophytes that represent potential candidates for future investigations in the context of host-enhancement effects and biocontrol applications. 

Species in the genus, *Cladosporium*, are common endophytes also found on conifer hosts [65,66]. The *Cladosporium* sp. identified in this study (sample Jeff56) also appears to impart a benefit to its host (*P*. *jeffreyi*) as it was the most successful antagonist against *H. parviporum* in this study (Table 4; Figure 5). The fungal endophyte, *Clonostachys* sp., was identified in the roots of *Picea glauca*. While there are no known instances of this specific root endophyte–host pairing, *Clonostachys* itself is a well-known root endophyte genus with some species imparting potential benefits to the host plant [67,68]. However, in this study, it was not a successful antagonist against the root rot pathogen (Table 4). Interestingly, several occurrences of the Cylindrocarpon-like pathogenic fungus, *Dactylonectria* sp. (best match *Dactylonectria torresensis*), were found in this study (in *P*. *glauca* and *P*. *peuce*, respectively), in which the sample, Glauc13, was one of the successful antagonists of *H. parviporum*. This fungus has been reported as the cause of root rot and decline in host health in strawberry, raspberry, olive trees, and in apple orchards [69,70,71]. Although species from the genus, *Dactylonectria*, have been encountered in European old-growth forests [72], no study has reported the presence of this genus in *Picea* or *Pinus* before. *Fusarium* spp. were recovered from multiple hosts (Table 2). *Fusarium* is a well-documented and ubiquitous root endophyte-containing genus [73,74,75] of which some species are pathogenic upon their hosts [76]. The results obtained in this study demonstrated that some *Fusarium* strains proved to be successful in the antagonism assay (Table 4). The Ilyonectria was also encountered in this study (in *P*. *omorika*). This is a widely-occurring genus of endophytic fungi [77,78,79,80] and at least one member has also been found in *P*. *glauca* [81]. Thus, this study is also likely the first time a member of this genus has been recorded in *P*. *omorika*. Ilyonectria spp. are also among frequently occurring non-DSE root endophytes. Whether the presence of these fungi in the hosts from this study is pathogenic or beneficial has yet to be defined. The results from this study indicates that the Ilyonectria (isolate Omori08) is a good antagonist of *H. parviporum*, suggesting that it could compete for niche space with other fungi [82].

*Penicillium* is a genus of well-known root endophytes that include non-DSE species found in grasses [83], orchids [67], and woody plants [84]. *Penicillium* spp. are also encountered in the roots of conifer hosts [85]. One *Penicillium* spp. (sample Omori01) proved to be a successful antagonist against *H. parviporum* (Table 4). This sample’s closest *Penicillium* relative is *P. glandicola*, which was only recently revealed as an endophyte in the cactus, *Opunita ficus-indica* [86]. *Pseudogymnoascus* sp. in this study was the only sample isolated from *P*. *peuce*. *Pseudogymnococcus* spp. are well-known root endophytes recovered from a variety of hosts, including Ericaceae, *Pinus*, and *Picea* [87,88,89]. *Pyrenochaeta* is a genus of fungi that contains several DSEs in grasses [83], shrubs [90], and woody species [42,62]. Although a *Pyrenochaeta* species was reported as an endophyte evoking inhibition of growth in the ash dieback pathogen, *Hymenoscyphus fraxineus* [91]—indicating that it can be a beneficial endophyte to its host—it has never before been reported as a conifer endophyte. *Pyrenochaeta* spp. have only been reported in conifers in the role of fungal decomposers of dead wood [92]. In this study, not only *Pyrenochaeta* spp. have been found in the roots of multiple host species (*P*. *abies*, *P*. *glauca*, *P*. *sitchensis*, and *Pinus jeffreyi*, Table 2), but we also observed they can be successful antagonists against *H*. *parviporum* (Table 4). The *Pyrenochaeta* sp. (Glauc18 and Sitch48) exhibited statistically significant antagonism on both day 7 and day 10 of measurement (Table 4). Thus, the genus, *Pyrenochaeta*, may hold interesting possibilities for the biocontrol of pathogenicity in conifer hosts. *Trichoderma* is a genus of well-known endophytes found in orchids [93], herbaceous plants [94], woody species [95], and conifer hosts [96]. *Trichoderma* sp. were found in this study from *P*. *sylvestris*. None of the isolates, however, were an effective antagonist against *H*. *parviporum*, indicating that *Trichoderma* in the roots does not play a defensive role against this pathogen. Pezizales sp. observed in this study are also a known root endophyte of conifer hosts as at least one member of this order has been encountered in a conifer previously [62]. 

Molecular phylogenetic analysis by maximum likelihood (ML) based on concatenated ITS sequences (Figure 4) suggests that the unidentified fungal sp. (Omori03) is likely a close relative of Pseudogymnoascus, indicating that this fungus might belong to the Ascomycetes class. Unfortunately, the node support for the branches from which these relationships were inferred is <90%, so a final conclusion in the absence of other corroborating data cannot be made. This isolate, fungal sp. (Omori03), was one of the strongest antagonists against *H. parviporum* as it displayed statistically significant antagonism on both day 7 and day 10 of measurement (Table 4). The only PAC species was isolated from the host, *P*. *omorika*. The screened literature does not contain any records of the PAC occurring in *P. omorika*, making our result the first confirmation of the species complex’ presence in the roots of this conifer species. This PAC species was also found to be a strong antagonist against *H*. *parviporum*. With regards to PAC species as antagonist candidates, the PAC species identified in this study (Omori07) did offer above-average antagonism to *H*. *parviporum*, but only on day 10 of measurement (Table 4). 

In theory, assuming strength by variety and numbers, *P. jeffreyi* with nine successful root endophytic antagonists would be the best protected tree species against *H*. *parviporum*. Hence, we can assume a possible correlation between hosting a greater number of antagonist endophytes and level of defensive capabilities against root pathogens. This, however, remains a hypothesis and requires validation for further confirmation, especially as *P. jeffreyi* is not the main host tree of *H. parviporum* [35]). 

Root fungal endophytes are important co-inhabitants of the rhizosphere of coniferous host species. This study has identified a variety of DSE and non-DSE root endophytes from coniferous hosts, including undiscovered root fungal endophyte diversity (e.g., in *P*. *omorika*). The antagonism assays performed indicate that potential antagonist root endophytes against *H*. *parviporum* can be found in a wide variety of coniferous hosts, and that some of these antagonists may also be suitable for long-term biocontrol of the pathogen due to their temporally stable and persistent antagonism. The results of this study also indicate that *Cladosporium* sp. and *Pyrenochaeta* sp. are very strong antagonists to *H*. *parviporum* under laboratory conditions in different ways. *Cladosporium* sp. did not exhibit temporally persistent antagonism, and while *Pyrenochaeta* sp. exhibited temporally persistent antagonism, it was not the strongest antagonist. Results established in this paper and the surveyed literature demonstrate that the endophyte–host interactions are complex and affect a wide variety of interactions [5,97], and that even pathogenic fungi (e.g., *Dactylonectria*) can act as successful antagonists against other pathogens (e.g., *H. parviporum*), offering protection to the colonized hosts in the process. Schlegel et al. [98] demonstrated that there is probably no clear-cut distinction between a pathogenic endophyte and a mutualistic one, with the endophyte likely switching roles seamlessly as environmental and host-associated factors vary [99]. The factors affecting the antagonist–pathogen relationship also need to be fully elucidated. In addition, the mechanisms mediating these interactions are also not fully understood nor generalized [62]. It was only recently demonstrated experimentally that root endophytes safeguard hosts from root pathogens by quickly occupying the newly growing roots [8]. In this context, antagonistic endophytes as biocontrol measures offer an enticing alternative to chemical methods in forestry [5]. Successful use of root fungal endophytes as biocontrol agents would thus require a thorough understanding of the endophyte–host relationship, the endophyte–pathogen relationship, the host–pathogen relationship, and the influence of environmental factors on these relationships.

Only six of the 65 root endophyte samples displayed statistically significant antagonism on both time points, 7 and 10 days, of the assay. This is indicative of a temporal aspect to the antagonism under this study’s experimental conditions—namely, that in a purely 1 vs. 1 scenario with limited space and nutrient availability, a root endophyte might be initially effective against a pathogen only for its antagonistic ability to falter later where the pathogen is potentially the better competitor/survivor under a more resource-constrained situation. With this in mind, selection of endophytes for potential biocontrol applications should then take into account the temporal aspects of the antagonism offered by the fungus. The temporal aspects primarily relate to the instantaneous stability of the antagonism (i.e., there must be minimal variation in the measurable antagonistic effects against the pathogen over time) and to the persistence of the antagonistic effect (i.e., the antagonistic effect must not taper off with time). In the experimental context, this implies that promising candidates from initial, short-term antagonism screenings should then be subjected to extended screening (preferably over multiple years) under lab and field conditions.

## 5. Conclusions

Root endophytes are effective antagonists against *H. parviporum*, suggesting that they may be of benefit to their host tree(s), should these endophytes form either mutualistic or commensalistic relationships with the host tree. On the other hand, two types of pathogenic fungi were also recovered as root endophytes, with some occurrences of them actually being good antagonists against *H. parviporum* (Table 4). Further, it is also possible that the sampled trees host entirely different root endophytes compared to their forest counterparts, as these were old, mature trees situated in a botanical garden. Nonetheless, these results imply that there is no simplistic picture of the root endophyte–host tree relationship, and that these interactions need to be investigated in detail with a more all-encompassing range of methodologies on a case-by-case basis. The mechanistic implications of the presence of the discovered root endophytes upon their respective hosts are beyond the scope of this study. In conclusion, this study identified the root fungal endophytes of several conifer hosts, which demonstrated successful antagonism against the white rot pathogen, *H. parviporum*. The data, conclusions, and inferences generated by this study will serve as the basis for executing future investigations into their potential for acting as biocontrol agents against root pathogens.

## Figures and Tables

**Table 1:** List of sampled host conifers, their common names, native ranges, and sample abbreviations used in the data.

Host Species	Common Name	Native to	Abbreviation
*Picea abies*	Norway spruce	Northern, Central, and Eastern Europe	Abie
*Picea glauca*	White/Canadian spruce	Alaska through central Canada to Newfoundland	Glau
*Picea omorika*	Serbian spruce	Endemic to Drina river valley, Serbia	Omori
*Picea pungens*	Blue/Colorado spruce	Rocky Mountains, USA	Pung
*Picea sitchensis*	Sitka spruce	West coast Canada, down to California	Sitch
*Pinus jeffreyi*	Jeffrey pine	Oregon-California, USA	Jeff
*Pinus peuce*	Macedonian/Balkan pine	Mountains of Balkan region	Peuc
*Pinus sylvestris*	Scots pine	Eurasia	Sylv

**Table 2:** The isolated fungal root endophytes’ frequency by tree host species.

Identification	Best Match Accession no.	Order	Class	*Picea*				*Pinus*			Sum
				*abies*	*glauca*	*omorika*	*sitchensis*	*jeffreyi*	*peuce*	*sylvestris*	
*Pseudogymnoascus* sp.	MH857254	Incertae sedis	Ascomycetes						1		1
*Cladosporium* sp.	MH863129	Capnodiales	Dothideomycetes					2			2
*Pyrenochaeta* sp.	KT269928	Pleosporales	Dothideomycetes	2			4				6
*Pyrenochaeta* sp.	LT821390	Pleosporales	Dothideomycetes		1			7			8
*Penicillium* sp.	MG821367	Eurotiales	Eurotiomycetes			1					1
*Penicillium* sp.	MH865458	Eurotiales	Eurotiomycetes			1					1
PAC ^b^	AY606286	Helotiales	Leotiomycetes			1					1
Fungal sp.	KY322665	NA	NA			2					2
Pezizales sp.	MH859398	Pezizales	Pezizomycetes							1	1
*Clonostachys* sp.	KY977560	Hypocreales	Sordariomycetes	3							3
*Dactylonectria* sp.	MH865183	Hypocreales	Sordariomycetes		8				1		9
*Fusarium* sp.	MG252283	Hypocreales	Sordariomycetes			2		6			8
*Fusarium* sp.	MG704912	Hypocreales	Sordariomycetes	2							2
*Ilyonectria* sp.	MH865177	Hypocreales	Sordariomycetes	1		2					3
*Trichoderma* sp.	MH930456	Hypocreales	Sordariomycetes							5	5
*Unknown*		NA	NA				6				6
*Unknown*		NA	NA					2			2
*Unknown*		NA	NA					1			1
*Unknown*		NA	NA	3							3
			**TOTAL**	11	9	9	10	18	2	6	**65**

^b^ PAC: *Phialocephala fortinii* s.l.—*Acephala applanata* species complex.

**Table 3:** Sequenced root endophytes and their best matches in the National Center for Biotechnology Information (NCBI) database based on concatenated internal transcribed spacer (ITS1-ITS2) sequences. The samples are grouped according to host tree species, and the GenBank accession numbers, sequence similarity (maximum identity/query coverage), as well as region of the homologous sequences used to establish molecular identity are shown.

Sample ID	Host	Best Match	Accession	Mi/Qc ^a^	Region	Our Definition
Abie23	*Picea abies*	*Clonostachys candelabrum*	KY977560	100/100	Croatia	*Clonostachys* sp.
Abie26	*Picea abies*	*Fusarium tricinctum*	MG704912	100/099	S. Korea	*Fusarium* sp.
Abie27	*Picea abies*	*Pyrenochaeta sp.*	KT269928	099/100	Greece	*Pyrenochaeta* sp.
Glau13	*Picea glauca*	*Dactylonectria torresensis*	MH865183	098/100	Portugal	*Dactylonectria* sp.
Glau17	*Picea glauca*	*Dactylonectria torresensis*	MH865183	099/100	Portugal	*Dactylonectria* sp.
Glau18	*Picea glauca*	*Pyrenochaeta inflorescentiae*	LT821390	099/100	Germany	*Pyrenochaeta* sp.
Glau21	*Picea glauca*	*Dactylonectria torresensis*	MH865183	100/100	Portugal	*Dactylonectria* sp.
Jeff49	*Pinus jeffreyi*	*Fusarium solani*	MG252283	099/100	China	*Fusarium* sp.
Jeff51	*Pinus jeffreyi*	*Pyrenochaeta inflorescentiae*	LT821390	099/100	Germany	*Pyrenochaeta* sp.
Jeff56	*Pinus jeffreyi*	*Cladosporium subinflatum*	MH863129	099/100	Slovenia	*Cladosporium* sp.
Jeff59	*Pinus jeffreyi*	*Pyrenochaeta inflorescentiae*	LT821390	099/100	Germany	*Pyrenochaeta* sp.
Jeff60	*Pinus jeffreyi*	*Pyrenochaeta inflorescentiae*	LT821390	099/100	Germany	*Pyrenochaeta* sp.
Jeff61	*Pinus jeffreyi*	*Pyrenochaeta inflorescentiae*	LT821390	099/100	Germany	*Pyrenochaeta* sp.
Jeff65	*Pinus jeffreyi*	*Pyrenochaeta inflorescentiae*	LT821390	099/100	Germany	*Pyrenochaeta* sp.
Omori01	*Picea omorika*	*Penicillium glandicola*	MG821367	099/100	Italy	*Penicillium* sp.
Omori02	*Picea omorika*	*Penicillium granulatum*	MH865458	099/100	USA	*Penicillium* sp.
Omori03	*Picea omorika*	Fungal sp. KK15	KY322665	099/100	Montenegro	NA
Omori07	*Picea omorika*	*Phialocephala fortinii*	AY606286	099/100	Sweden	PAC ^b^
Omori08	*Picea omorika*	*Ilyonectria lusitanica*	MH865177	099/100	Portugal	*Ilyonectria* sp.
Peuc68	*Pinus peuce*	*Dactylonectria torresensis*	MH865183	100/100	Portugal	*Dactylonectria* sp.
Peuc69	*Pinus peuce*	*Pseudogymnoascus pannorum*	MH857254	100/100	Germany	*Pseudogymnoascus* sp.
Sylv36	*Pinus sylvestris*	*Trichoderma koningii*	MH930456	099/100	Spain	*Trichoderma* sp.
Sylv37	*Pinus sylvestris*	*Trichoderma koningii*	MH930456	099/100	Spain	*Trichoderma* sp.
Sylv38	*Pinus sylvestris*	*Ascobolus lineolatus*	MH859398	097/099	Tanzania	Pezizales sp.

^a^ Mi/Qc: Match identity (%)/Query coverace (%); ^b^ PAC: *Phialocephala fortinii s.l.* – *Acephala applanata* species complex.

**Table 4:** Root endophytes identified as successful antagonists against the pathogen, *H. parviporum*, in the antagonism assay. These samples were identified on the basis of the significance of a t-test (*p*-value = 0.05) comparing the mean spherical index of their pathogen partners (*n* = 3 trials) with the mean spherical index of the pathogen controls (*n* = 12).

Sample ID	Host	Identification	Sampling Time (days) ^a^		
			3	7	10
Abie25	*Picea abies*	*Fusarium* sp.	N	Y	N
Abie29	*Picea abies*	Unknown	N	N	Y
Glauc15	*Picea glauca*	*Dactylonectria* sp.	N	Y	Y
Glauc19	*Picea glauca*	*Dactylonectria* sp.	N	N	Y
Glauc13	*Picea glauca*	*Dactylonectria* sp.	N	Y	N
Glauc18	*Picea glauca*	*Pyrenochaeta* sp.	N	Y	Y
Omori01	*Picea omorika*	*Penicillium* sp.	N	Y	N
Omori03	*Picea omorika*	Fungal sp.	N	Y	Y
Omori07	*Picea omorika*	PAC ^b^	N	N	Y
Omori08	*Picea omorika*	*Ilyonectria* sp.	N	Y	N
Omori06	*Picea omorika*	Fungal sp.	N	Y	N
Jeff50	*Pinus jeffreyi*	*Fusarium* sp.	N	Y	Y
Jeff55	*Pinus jeffreyi*	*Fusarium* sp.	N	N	Y
Jeff58	*Pinus jeffreyi*	*Fusarium* sp.	N	N	Y
Jeff62	*Pinus jeffreyi*	*Pyrenochaeta* sp.	N	N	Y
Jeff63	*Pinus jeffreyi*	*Pyrenochaeta* sp.	N	N	Y
Jeff64	*Pinus jeffreyi*	Unknown	N	N	Y
Jeff51	*Pinus jeffreyi*	*Pyrenochaeta* sp.	N	Y	N
Jeff56	*Pinus jeffreyi*	*Cladosporium* sp.	N	Y	N
Jeff65	*Pinus jeffreyi*	*Pyrenochaeta* sp.	N	N	Y
Sitch40	*Pinus sitchensis*	*Pyrenochaeta* sp.	N	N	Y
Sitch41	*Pinus sitchensis*	*Pyrenochaeta* sp.	N	N	Y
Sitch42	*Pinus sitchensis*	Unknown	N	N	Y
Sitch46	*Pinus sitchensis*	Unknown	N	Y	Y
Sitch47	*Pinus sitchensis*	*Pyrenochaeta* sp.	N	N	Y
Sitch48	*Pinus sitchensis*	*Pyrenochaeta* sp.	N	Y	Y

^a^ ‘Y’ is statistically significant, ‘N’ is insignificant (for *p*-value = 0.05). ^b^ PAC: *Phialocephala fortinii* s.l.—*Acephala applanata* species complex.

## Supplementary Materials

The following are available online at https://www.mdpi.com/2076-2607/7/4/102/s1. Word file S1: #R script for identification of successful conifer root-associated fungal endophyte antagonists against *Heterobasidion parviporum* based on in vitro dual-culture assays, Excel file S2: New R-based methodology to optimize the identification of root endophytes against *Heterobasidion parviporum*.
