# Supplementary material for: New R-Based Methodology to Optimize the Identification of Root Endophytes against Heterobasidion parviporum
Source: Microorganisms. 2019 Apr 6;7(4):102. doi: 10.3390/microorganisms7040102 (PMC6517935; doi:10.3390/microorganisms7040102)
Supplement: Supplementary file 1 [file microorganisms-07-00102-s001.zip › Supplementary file 1.docx]

#R script for identification of successful conifer root-associated fungal endophyte antagonists against Heterobasidion parviporum based on in vitro dual-culture assays

#Authors - Linda Rigerte, Kathrin Blumenstein, Eeva Terhonen (Georg-August-Universitaet Goettingen)

#Begin code

#Index of common terminology used in this script

#Fab - spherecity index of root fungal endophyte (root FE)

#Pab - spherecity index of pathogen (H. parviporum)

#FabMean/FabSD - average and standard deviation (respectively) of root FE samples (n=3)

#PabMean/PabSD - average and standard deviation of pathogen samples (n=3)

#Packages used + descriptions thereof

#For Excel raw file import/export

library(openxlsx)

#For miscellaneous tools

library(data.table)

#For miscellaneous tools

library(dplyr)

#For scatterplot3d()

library(scatterplot3d)

#For plot3d()

library(rgl)

#Changing working directory if necessary; note - all requisite pre-processed data files (.xlsx files) have to be placed in the appropriate working directory as indicated in this script

#getwd()

#setwd()

#For methods section - simulated plot to show data reduction step

Pab <- rnorm (100, mean = 1, sd = 0.5)

Fab <- rnorm (100, mean = 1, sd = 0.5)

plot(Pab~Fab, xlab = "Mean sphericity index for antagonist fungal endophyte", ylab = "Mean sphericity index for antagonist pathogen", xlim = c(0, 2.0), ylim = c(0, 2.0), col= ifelse(Pab < 1 & Fab > 1, "green", "red"), pch=20)

abline(0,1)

abline(h = 1, v = 1)

#Import data from Excel - note some data preprocessing done manually in Excel

tab0 <- read.xlsx("book1.xlsx")

head(tab0)

#Import raw data without the precalculated, pre-filtered means in tab0

tab1 <- read.xlsx("book2.xlsx")

head(tab1)

#Calculate means and SD

#Fungus spherecity mean and SD

tab1$FabMean <- apply(tab1[,5:7], 1,mean)

tab1$FabSD <- apply(tab1[, 5:7], 1, sd)

#Pathogen spherecity mean

tab1$PabMean <- apply(tab1[,8:10], 1,mean)

tab1$PabSD <- apply(tab1[, 8:10], 1, sd)

#Remove all samples from tab1 where FabMean and PabMean are NA in Timedays = 3, 7, and 10; using the tab0 set as guide for this

#tab2 <- tab1[((!is.na(tab1$FabMean) & !is.na(tab1$PabMean) & tab1$Timedays == 3) & (!is.na(tab1$FabMean) & !is.na(tab1$PabMean) & tab1$Timedays == 7) & (!is.na(tab1$FabMean) & !is.na(tab1$PabMean) & tab1$Timedays == 10)),]

tab2 <- subset(tab1, tab1$SampID %in% tab0$SampID)

#First plot PabMean vs FabMean (y~x) to investigate for which of the samples sphericity index for the fungus is >1 AND ALSo the spherecity index for the pathogen is <1

#These are the only cases where the fungus has definitely behaved antagonistically against the pathogen

#All other samples can be considered spurious instances and can be discarded

#Write these out as separate subsets

#All samples

with(tab2, plot(PabMean~FabMean, xlim = c(0.5, 1.5), ylim = c(0.5, 1.5)))

abline(a = 0.00, b = 1)

abline(h = 1, v = 1)

#Day 3 subset

with(tab2[which(tab2$Timedays == 3),], plot(PabMean~FabMean, xlim = c(0.5, 1.5), ylim = c(0.5,1.5), asp = 1))

abline(h = 1, v = 1)

tab3 <- subset(tab2, tab2$PabMean < 1 & tab2$FabMean > 1 & tab2$Timedays == 3)

with(tab3, plot(PabMean~FabMean, xlim = c(0.5, 1.5), ylim = c(0.5,1.5), asp = 1))

abline(h = 1, v = 1)

#Day 7 subset

with(tab2[which(tab2$Timedays == 7),], plot(PabMean~FabMean, xlim = c(0.5, 1.5), ylim = c(0.5,1.5), asp = 1))

abline(h = 1, v = 1)

tab7 <- subset(tab2, tab2$PabMean < 1 & tab2$FabMean > 1 & tab2$Timedays == 7)

with(tab7, plot(PabMean~FabMean, xlim = c(0.5, 1.5), ylim = c(0.5,1.5), asp = 1))

abline(h = 1, v = 1)

#Day 10 subset

with(tab2[which(tab2$Timedays == 10),], plot(PabMean~FabMean, xlim = c(0.5, 1.5), ylim = c(0.5,1.5), asp = 1))

abline(h = 1, v = 1)

tab10 <- subset(tab2, tab2$PabMean < 1 & tab2$FabMean > 1 & tab2$Timedays == 10)

with(tab10, plot(PabMean~FabMean, xlim = c(0.5, 1.5), ylim = c(0.5,1.5), asp = 1))

abline(h = 1, v = 1)

#Transposing the datasets from the replicates in rows to replicates in columns

tab3T <- melt(setDT(tab3), measure=patterns("FR", "PR"), variable.name="Replicates", value.name=c("FRab", "PRab"))[order(SampID)][, Replicates := paste0("Rep", Replicates)][]

head(tab3T)

tab7T <- melt(setDT(tab7), measure=patterns("FR", "PR"), variable.name="Replicates", value.name=c("FRab", "PRab"))[order(SampID)][, Replicates := paste0("Rep", Replicates)][]

head(tab7T)

tab10T <- melt(setDT(tab10), measure=patterns("FR", "PR"), variable.name="Replicates", value.name=c("FRab", "PRab"))[order(SampID)][, Replicates := paste0("Rep", Replicates)][]

head(tab10T)

#Importing pathogen ctrl data as a separate dataframe

pathtab <- read.xlsx("Book3.xlsx")

pathtab3 <- subset(pathtab, pathtab$Timedays == 3)

pathtab7 <- subset(pathtab, pathtab$Timedays == 7)

pathtab10 <- subset(pathtab, pathtab$Timedays == 10)

#Statistics

#Day3 samples

#Compare first all Pab values from particular tree against P-ctrl values

tab3Ta <- tbl_df(tab3T)

res3 <- tab3Ta %>% group_by(TreeSp) %>%

do(tpvals = t.test(.$PRab, pathtab3$Pctrlab, alternative="less", paired = FALSE, var.equal = FALSE)) %>%

summarise(TreeSp, pvals = tpvals$p.value)

res3

#Group by TreeSp AND HavDNA

res3 <- tab3Ta %>% group_by(TreeSp, HavDNA) %>%

do(tpvals = t.test(.$PRab, pathtab3$Pctrlab, alternative="less", paired = FALSE, var.equal = FALSE)) %>%

summarise(TreeSp, HavDNA, pvals = tpvals$p.value)

res3

#Every individual sample

res3 <- tab3Ta %>% group_by(TreeSp, SampID, HavDNA) %>%

do(tpvals = t.test(.$PRab, pathtab3$Pctrlab, alternative="less", paired = FALSE, var.equal = FALSE)) %>%

summarise(TreeSp, SampID, HavDNA, pvals = tpvals$p.value)

res3

res3a <- res3[which(res3$pvals < 0.05),]

#Plot and data out <- no significant results so code is commented out

#tab3b <- subset(tab3, tab3$SampID %in% res3a$SampID)

#with(tab3b, plot(PabMean~FabMean, xlim = c(0.5, 1.5), ylim = c(0.5, 1.5)))

#text(tab3b$PabMean, tab3b$FabMean, labels = tab3b$SampID, cex = 0.7, pos = 1)

#abline(a = 0.00, b = 1)

#abline(h = 1, v = 1)

#write.xlsx(tab3b, "3daysigs.xlsx")

#Day 7 samples

#Compare first all Pab values from particular tree against P-ctrl values

tab7Ta <- tbl_df(tab7T)

res7 <- tab7Ta %>% group_by(TreeSp) %>%

do(tpvals = t.test(.$PRab, pathtab7$Pctrlab, alternative="less", paired = FALSE, var.equal = FALSE)) %>%

summarise(TreeSp, pvals = tpvals$p.value)

res7

#Group by TreeSp AND HavDNA

res7 <- tab7Ta %>% group_by(TreeSp, HavDNA) %>%

do(tpvals = t.test(.$PRab, pathtab7$Pctrlab, alternative="less", paired = FALSE, var.equal = FALSE)) %>%

summarise(TreeSp, HavDNA, pvals = tpvals$p.value)

res7

#Every individual sample

res7 <- tab7Ta %>% group_by(TreeSp, SampID, HavDNA) %>%

do(tpvals = t.test(.$PRab, pathtab7$Pctrlab, alternative="less", paired = FALSE, var.equal = FALSE)) %>%

summarise(TreeSp, SampID, HavDNA, pvals = tpvals$p.value)

res7

res7a <- res7[which(res7$pvals < 0.05),]

#Plot and data out

tab7b <- subset(tab7, tab7$SampID %in% res7a$SampID)

with(tab7b, plot(PabMean~FabMean, xlim = c(0.5, 1.5), ylim = c(0.5, 1.5)))

#text(tab7b$PabMean, tab7b$FabMean, labels = tab7b$SampID, cex = 0.7, pos = 1)

abline(h = 1, v = 1)

write.xlsx(tab7b, "7daysigs.xlsx")

#Day 10 samples

#Compare first all Pab values from particular tree against P-ctrl values

tab10Ta <- tbl_df(tab10T)

res10 <- tab10Ta %>% group_by(TreeSp) %>%

do(tpvals = t.test(.$PRab, pathtab10$Pctrlab, alternative="less", paired = FALSE, var.equal = FALSE)) %>%

summarise(TreeSp, pvals = tpvals$p.value)

res10

#Group by TreeSp AND HavDNA

res10 <- tab10Ta %>% group_by(TreeSp, HavDNA) %>%

do(tpvals = t.test(.$PRab, pathtab10$Pctrlab, alternative="less", paired = FALSE, var.equal = FALSE)) %>%

summarise(TreeSp, HavDNA, pvals = tpvals$p.value)

res10

#Every individual sample

res10 <- tab10Ta %>% group_by(TreeSp, SampID, HavDNA) %>%

do(tpvals = t.test(.$PRab, pathtab10$Pctrlab, alternative="less", paired = FALSE, var.equal = FALSE)) %>%

summarise(TreeSp, SampID, HavDNA, pvals = tpvals$p.value)

res10

res10a <- res10[which(res10$pvals < 0.05),]

#Plot and data out

tab10b <- subset(tab10, tab10$SampID %in% res10a$SampID)

with(tab10b, plot(PabMean~FabMean, xlim = c(0.5, 1.5), ylim = c(0.5, 1.5)))

#text(tab10b$PabMean, tab10b$FabMean, labels = tab10b$SampID, cex = 0.7, pos = 1)

abline(h = 1, v = 1)

write.xlsx(tab10b, "10daysigs.xlsx")

#Samples found in both day 7 and day 10 that were statistically significant; boxplots

library(lattice)

#7day data

tabm1 <- subset(tab7b, tab7b$SampID %in% tab10b$SampID)

#10day data

tabm2 <- subset(tab10b, tab10b$SampID %in% tab7b$SampID)

#Rowbind both

tabm <- rbind(tabm1, tabm2)

#Simple day 7 and day 10 boxplot

with(tabm, boxplot((PabMean/FabMean)~Timedays, ylim = c(0.5, 1.0)))

#Melt columns to get raw values in single columns under FR and PR again

temp <- melt(setDT(tabm), measure=patterns("FR", "PR"), variable.name="Replicates", value.name=c("FRab", "PRab"))[order(SampID)][, Replicates := paste0("Rep", Replicates)][]

head(temp)

#Boxplot - unit change in sphericity index of pathogen per unit change in sphericity index of root FE by sample by time for all samples that were statistically significant in day 7 and day 10

bwplot((PRab/FRab) ~ SampID | paste0("Time (days) ", Timedays), data = temp, as.table = TRUE, index.cond=list(c(2, 1)), xlab = "Sample", ylab = "Sphericity index ratio (Pathogen/Root FE)")

#Paired t-test to compare change in the above means from day 7 to day 10

#All samples

with(temp, t.test(PRab[Timedays==7]/FRab[Timedays==7], PRab[Timedays==10]/FRab[Timedays==10], paired=TRUE))

#Sample by sample

restemp <- temp %>% group_by(TreeSp, SampID, HavDNA) %>%

do(tpvals = t.test(.$PRab[.$Timedays==7]/.$FRab[.$Timedays==7], .$PRab[.$Timedays==10]/.$FRab[.$Timedays==10], paired=TRUE)) %>%

summarise(TreeSp, SampID, HavDNA, pvals = tpvals$p.value)

restemp

restemp <- restemp[which(restemp$pvals < 0.05),]

#3d scatterplot to visualize the Pab/Fab change over time!

#with(temp, scatterplot3d(Timedays, FabMean, PabMean, xlim=c(7,10),ylim=c(0,2), zlim=c(0,2)))

with(temp, plot3d(Timedays, FabMean, PabMean, xlim=c(5,11),ylim=c(0,2), zlim=c(0,2), xlab="Time (days)", ylab="Root FE", zlab="Pathogen"))

#End code
